# Enrolling and retaining a representative population in the NIH Researching COVID to Enhance Recovery (RECOVER)-Adult Cohort

**DOI:** 10.21203/rs.3.rs-10092514/v1

**Published:** 2026-07-06

**Authors:** Jasmine Berry, Jennifer C. Gander, Jun Lu, Franchesca Amor A. Aguilar, Larissa Teunis, A. Blythe Ryerson, Sarah de Ramirez, Sarah E. Donohue, Christina Mehta, Jenny E. Han, Sushma K. Cribbs, Tiffany A. Walker, Yasha Joseph, Priscilla Pemu, Vincent C. Marconi, Zanthia Wiley, Monica Verduzco-Gutierrez, Ingrid V. Bassett, Nasser Sharareh, Igho Ofotokun, Marissa Edmiston, Vidhi Javia, Jannah Elchommali, Cynthia Ifejika, Ke'Ara Brown-Smith, Walter Asencios, Imani Elsey, Samer Sader, John W. Hafner, Monica Hendrickson, Sara W. Kelly, Sairam Parthasarathy, Kristen Pogreba-Brown, Perla Nunes, Leah Castro, Doreen Stein-Seroussi, Kian Nguyen, Robynn Saldino, Janelle Linton, Stacey Riddick, Jasmine Briscoe, Jerry A. Krishnan, Lynn B. Gerald

**Affiliations:** Emory University School of Medicine; Centre College; University of Illinois Chicago; Emory University School of Medicine; University of New Mexico; Kaiser Permanente Georgia; Illinois Research Network (ILLInet) Hub, OSF HealthCare; Illinois Research Network (ILLInet) Hub, OSF HealthCare; Emory University School of Medicine; Emory University School of Medicine, Grady Memorial Hospital; Emory University School of Medicine, Grady Memorial Hospital; Emory University School of Medicine; Emory University, Emory College of Arts and Sciences; Morehouse School of Medicine; Emory University School of Medicine; Emory University School of Medicine; University of Texas Health Science Center at San Antonio Hub, University of Texas Health Science Center at San Antonio; Massachusetts General Hospital; University of Utah Hub, University of Utah; Case Western University Hub, Metrohealth Medical Center; Case Western University Hub, Metrohealth Medical Center; Emory University School of Medicine, Grady Memorial Hospital; Emory University School of Medicine, Grady Memorial Hospital; Emory University School of Medicine, Grady Memorial Hospital; Emory University School of Medicine, Grady Memorial Hospital; Emory University School of Medicine, Grady Memorial Hospital; Emory University School of Medicine, Grady Memorial Hospital; University of Illinois Chicago; Illinois Research Network (ILLInet) Hub, OSF HealthCare; Illinois Research Network (ILLInet) Hub, OSF HealthCare; Illinois Research Network (ILLInet) Hub, OSF HealthCare; University of Arizona Hub, University of Arizona; University of Arizona Hub, University of Arizona; NYU School of Grossman; NYU School of Grossman; NYU School of Grossman; NYU School of Grossman; NYU School of Grossman; NYU School of Grossman; NYU School of Grossman; NYU School of Grossman; University of Illinois Chicago; University of Illinois Chicago

**Keywords:** COVID-19, recruitment, retention, diversity, Long COVID, epidemiology

## Abstract

**Background:**

Vulnerable populations are underrepresented in clinical studies. The design of the national RECOVER-Adult cohort study aimed to over-enroll underrepresented populations to ensure the representation of vulnerable populations in the U.S. We quantify and assess follow-up visit completion among a representative population in the RECOVER-Adult cohort.

**Methods:**

Between October 2021 and October 2023, we enrolled adult participants across 16 hub-sites in the U.S. Community outreach was used to gather and incorporate feedback prior to and during study implementation. Recruitment methods included electronic and in-person outreach by individuals trained in diversity, equity, and inclusion at health centers, health departments, and community-based organizations, as well as snowball sampling. Study staff maintained regular contact with participants between study visits via phone, email, and text, and arranged free transportation to support attendance.

**Results:**

Of the 14,880 adult participants who were enrolled in the RECOVER-Adult Cohort Study, 14,531 participants (mean[SD] age: 47.1[15.5] years, 57.0% Non-Hispanic White, 27.7% Male) completed a baseline visit. Over 85% of participants completed the first four follow-up visits, while over 90% completed a last visit. Across follow-up visits, participants who identified as minoritized groups, younger, LGBTQIA+, had less education, did not speak English, lived in a rural area, and reported a disability were less likely to complete their visits, while older and female participants were more likely to complete them.

**Conclusions:**

Our findings indicate that enrollment is more feasible than retaining a sociodemographically-representative study population, highlighting a need for innovative approaches to sustain on-going study participation in the post-pandemic era.

## Introduction

The difficulty of recruiting and retaining a representative cohort is underestimated (1–3). Disadvantaged populations, including racial and ethnic minority groups, may be apprehensive about participating in research studies due to experiences with medical racism, mistrust of medical research, healthcare provider bias, language barriers, and cultural alienation from researchers (1–3). In addition, lower educational attainment, disabilities, logistical barriers (e.g., transportation), and financial hardships can prevent participants from completing research studies (4, 5). Therefore, to maintain a representative and representative cohort it is necessary to proactively address these barriers to research.

The National Institutes of Health (NIH)-funded Researching COVID to Enhance Recovery (RECOVER) Initiative includes an Adult Cohort Study to understand the epidemiology of Long COVID, as well as the social and biologic factors that contribute to developing Long COVID among individuals aged 18 years and older (6). It is estimated that SARS-CoV-2 (COVID-19) has infected more than 750 million individuals globally, most of whom are adults, and has disproportionately impacted Black, Latino, and Indigenous American communities and individuals from lower socioeconomic backgrounds (6, 7). Long COVID (also known as post-acute sequelae of SARS-CoV-2 infection or PASC), is a newly identified infection-associated chronic condition that affects individuals for 3 or more months after SARS-CoV-2 infection and can affect any organ system (6). Estimates indicate that the cumulative incidence of Long COVID in the U.S. is about 20 million people and 400 million people worldwide (8). The design of the RECOVER-Adult Cohort Study called for enrollment of a representative sample to ensure that the findings are applicable to the general population in the U.S. (6). Several studies focused on chronic conditions have reported difficulty engaging a representative population, but there is a paucity of literature on targeted outreach and community-driven efforts in the postpandemic era. Thus, the objective of this analysis is to describe the sociodemographic characteristics and approaches to enroll and retain a representative participant population in the RECOVER-Adult Cohort Study, the largest prospective cohort study of Long COVID in the world.

## Methods

### Study Recruitment and Retention Methods

Detailed methods of the RECOVER study have been previously published and are expanded upon in the **Supplementary Materials** (9). Between October 2021 and October 2023, we enrolled adult participants across 86 enrolling sites that were managed by 16 hub-sites in 33 states in the U.S, including Washington, D.C., and Puerto Rico. The enrolling sites also included participants who were pregnant at the time of their SARS-CoV-2 infection or at the time of enrollment. The protocol for the pregnancy cohort has been described previously(10). Social media, websites, conventional mass media, secure encrypted emails, and flyers were utilized for recruitment, with all materials assessed for health literacy and IRB approval. Participants were identified via self-referral, community recruitment, and targeted outreach to specific populations, health system-based recruitment, and public health-based recruitment. For participants without SARS-CoV-2 infection, sites were asked to draw from communities, demographics, and care sites similar to those of the sites recruiting infected participants. Enrollment was confirmed by date of enrollment, race/ethnicity, sex (at birth), residence in rural or medically underserved areas, hospitalization status at time of initial infection, and referral source to allow for real-time adjustments in enrollment to match protocol targets. Clinical staff utilized text messaging, emails, phone calls, secure messages through the electronic health portal, automated messaging, and social media posts to communicate with participants about study updates and upcoming follow-ups. Some RECOVER sites offered transportation options to assist with participant recruitment and retention. After the enrollment visit, the follow-up schedule included questionnaires that could be completed online, every 3 months, and in-person study visits every 3 to 12 months depending on the time elapsed after the date of SARS-CoV-2 infection or the date of the negative test for SARS-CoV-2 in the control “uninfected” and the date of enrollment. Follow-ups occurred every 3 months and survey forms were available in English, Spanish, or Chinese. Participants were compensated monetarily for each in-person and virtual visit and, if selected, for additional lab assessments. Reimbursements for transportation costs and ride-share services were also provided at some sites to address financial and logistical issues participants had.

RECOVER also created community engagement groups composed of participant representatives who communicated concerns and issues from the patient’s perspective to the sites and who were involved in all aspects of study design, execution, and outcome dissemination.(11) Some of the recommendations from these community groups included ways to disseminate study results back to their communities, measures to extend enrollment for Hispanic and rural communities, improving survey forms to increase accessibility, creating plain language summaries of published research, creating study materials that are community-specific and culturally tailored, allowing community representatives to be a part of the manuscript development process, and inviting community representatives to research seminars and committees to share their experiences and give feedback directly to researchers.(12) Clinical research staff at some sites provided technology assistance to help complete online surveys and understand survey questions.

These analyses focused on the first four follow-up visits and the last follow-up visit that occurred within a year of the study’s end date (October 01, 2024 - October 31, 2025). We excluded participants who were non-enrolled, never started the study protocol, or were missing their index date. The index date is defined as the first positive SARS-COV-2 PCR, NAAT, or antibody test date or date of symptom onset for infected participants and the first negative test date for uninfected participants. Participants provided written informed consent. The NYU Grossman School of Medicine Institutional Review Board approved the study, in addition to local IRB approvals for certain sites.

### Outcome Definition

The primary outcomes were completion of the baseline visit, the first four follow-up visits after baseline, and the last visit that occurred within a year of the study’s end date (October 01, 2024 - October 31, 2025). A visit was determined to be completed if the participant attempted a symptom and social determinants of health questionnaire, an in-person study visit (e.g., biospecimen collection, smell test, non-contrast chest CT), or both, as specified in the study protocol as of October 31, 2025 (9). The last visit was considered complete if the participant completed a visit within 1 year of the study’s end date.

### Covariates

We measured the following sociodemographic variables based on participant-reported information: age in years at enrollment (18‒33, 34‒48, 49‒64, 65‒79, 80 years or older), race/ethnicity (Non-Hispanic White, Non-Hispanic Black, Non-Hispanic Asian, Hispanic, Two or more races, Other races (American Indian or Alaska Native, Native Hawaiian or other Pacific Islander, none of these fully describe me), sex assigned at birth (male, female/intersex), sexual orientation (straight, LGBTQIA+), education (high school or less, some college/trade school, college degree or higher), household income in 2019 (<$24,999, $25,000-$49,999, $50,000-$74,999, $75,000-$99,999, $100,000+), marital status (Divorced, widowed, separated, or never married, Married or living with partner), insurance status (yes/no), from a U.S. Health Services Administration's designated medically underserved area (yes/no), primary language (English, non-English), employment status (employed, unemployed), and disability status (yes/no), and reasons for withdrawal from the study (lost to follow-up, moved out of range of study, participant declines to participant further (with or without data retention), investigator withdrew participant, no longer eligible (e.g. incarcerated, cognitively impaired, etc.), deceased) (13). “Prefer not to answer” and “I don’t know” responses were included in missingness.

### Statistical Analysis

We calculated counts and percentages for baseline sociodemographic characteristics and by follow-up status at each visit (completed/not completed) and withdrawn status (yes/no). Differences in sociodemographic characteristics between follow-up status at each visit were compared using chi-square tests and Fisher’s exact test. We used logistic regression models to evaluate the association between each sociodemographic characteristic and visit completion status. The adjusted model included all sociodemographic characteristics. A secondary analysis was conducted to assess the association between sociodemographic characteristics and withdrawn status. Data were obtained from the RECOVER-Adult December 2026 data lock. All analyses were conducted in R Statistical Software (v4.5.2; R Core Team 2022). Significance level was set with a two-tailed p < 0.05 for all analyses.

## Results

Among the 14,880 participants enrolled in the RECOVER-Adult Cohort, 14,527 participants (mean[SD] age: 47.1[15.5] years, 57.0% Non-Hispanic White, 27.7% Male) completed a baseline study visit (Table 1). On average, participants had a study time of 29.1 months and completed approximately 9 follow-ups. Approximately 94.5% (n = 13,733) of participants completed at least 1 follow-up visit. Among participants who completed a visit, over half (54.9%, n = 7,538) completed 7–12 visits. (**Supplementary Table 1**). Follow-up visits 1, 2, 3, and 4 were completed by 89.9%, 87.3%, 84.7%, and 85.9% of participants, respectively. Participants who completed follow-up visit 1 (vs. missed) were older, on average, (47.4 years vs. 44.3 years, p < 0.001), more likely to have a college degree or higher (65.6% vs. 53.5%, p < 0.001), have a 2019 household income of $100,000 (37.3% vs. 28.5%, p < 0.001), be married or live with a partner (60.1% vs. 52.7%, p = 0.002), be employed (64.0% vs. 62.0%, p < 0.001), be from a medically underserved area (74.7% vs. 70.0%, p < 0.001), speak English as a primary language (90.7% vs. 83.5%, p = 0.001), not live in a rural area (95.9% vs. 94.0%, p < 0.001), and not report a disability (73.6% vs. 65.0%, p < 0.001) Similar trends were observed in follow-up visit 2, 3, and 4, with significance varying by marital status, from a medically underserved area, and living in a rural area. The last follow-up visit was completed by 91.8% of participants, with completion rates varying significantly by all characteristics except marital status, insurance status, being from a medically underserved area, primary language, and living in a rural area (Table 1). Additional details on the results by hub site are provided in the **Supplementary Materials**.

Approximately 21.7% (n = 3,149) of participants were withdrawn from the study. Participants who were withdrawn (vs. not withdrawn) were more likely to be younger (44.6 years vs. 47.8 years, p < 0.001), less likely to be non-Hispanic White (52.0% vs. 58.5%, p < 0.001), female (66.9% vs. 73.5%, p < 0.001), LGBTQIA+ (80.5% vs. 85.4%, p < 0.001), have a college degree or higher (55.0% vs. 66.5%, p < 0.001), have a 2019 household income of $100,000 (30.4% vs. 37.7%, p < 0.001), have insurance (87.3% vs. 92.8%, p = 0.008) and more likely to be unemployed (29.8% vs. 24.8% p < 0.001), from a medical underserved area (29.8% vs. 24.8%, p < 0.001), and report a disability (25.2% vs. 20.8%, p < 0.001) (Table 3). The majority of participants were withdrawn due to loss to follow-up (3 consecutive missed visits, n = 1,984, 63.0%) and declining to participate further (n = 887, 28.2%) (**Supplementary Table 1**).

### Association between follow-up visit completion and sociodemographic characteristics

For follow-up visit 1, in unadjusted models, participants had a lower odds of completing their follow-up visit if they were between the ages of 18–33 years (0.82 [0.72–0.95]), identified as non-Hispanic Black (0.57 [0.49–0.66]), Hispanic (0.63 [0.54–0.72]), or other races (0.49 [0.32–0.79]), had a high school education or less (0.42 [0.36–0.49]), some college/trade school education (0.76 [0.66–0.87]), had a 2019 household income of <$24,999 (0.58 [0.49–0.68]) or $25,000 - $49,999 (0.67 [0.56–0.79]), were divorced, widowed, separated, or never married (0.83 [0.74–0.93]), from a medically underserved area (0.79 [0.70–0.89]), did not speak English as a primary language (0.72 [0.59–0.88]), lived in a rural area (0.67 [0.53–0.85]), and reported a disability (0.80 [0.70–0.91]). Participants had a higher odds of completing their follow-up if they were between the ages of 49–64 years (1.20 [1.04–1.38]) or 65–79 years (1.58 [1.30–1.92]). In adjusted models, most associations were non-significant, with the exception of the 49–64 years age group (1.39 [1.14–1.69]), non-Hispanic Black (0.70 [0.55–0.89]), high school education or less (0.49 [0.38–0.65]), and living in a rural area (0.61 [0.44–0.88]) (Table 2).

For follow-up 2, patterns were similar to follow-up 1, with some exceptions. In unadjusted models, participants who identified as non-Hispanic Asian (0.66 [0.54–0.81]) and were unemployed (0.73 [0.56–0.97]) had a lower odds of completing their visit. Female participants had a higher odds (1.17 [1.05–1.31]) of completing their visit. In adjusted models, participants in the 18–33 year age group (0.74 [0.62–0.88]), identified as other races (0.40 [0.24–0.67]), and had some college/trade school education (0.81 [0.68–0.98]) had a lower odds of completing their visit while participants who were female (1.22 [1.04–1.42]), in the 65–79 year (1.51[1.09–2.16]) age group, and had a 2019 household income of $75,000-$99,999 (1.54 [1.21–1.97]) had a higher odds of completing the visit.

For follow-up 3, patterns were similar to follow-up 1, with some exceptions. In unadjusted models, participants who identified as non-Hispanic Asian (0.67 [0.55–0.81]) or LGBTQIA+ (0.84[0.73–0.97]) were less likely to complete their visit. Female participants were more likely (1.19 [1.07–1.31]) to complete their visit. In adjusted models, participants were less likely to complete their visit if they identified as non-Hispanic Asian (0.73 [0.57–0.95]) or other races (0.51 [0.31–0.88]) and had some college/trade school education (0.75 [0.63–0.88]). Participants were more likely to complete their visit if they were female (1.22 [1.06–1.41]) and in the 65–79 year age group (1.65 [1.21–2.31]).

For follow-up 4, patterns were similar to follow-up 1, with some exceptions. In unadjusted models, participants had a lower odds of completing their visit if they identified as non-Hispanic Asian (0.64 [0.53–0.78]), two or more races (0.69 [0.54–0.89]), or LGBTQIA+ (0.77 [0.67–0.89]). Participants were more likely to complete their visit if they were female (1.17 [1.05–1.30]). In adjusted models, participants were less likely to complete their visit if were in the 18–33 year age group (0.69 [0.58–0.81]), identified as non-Hispanic Asian (0.71 [0.55–0.93]) or other races (0.56 [0.33–0.99]). They were more likely to complete their visit if they were female (1.30 [1.12–1.51]) and in the 65–79 year age group (1.78 [1.27–2.57]).

For the last visit, findings were similar to follow up 1, with a few exceptions. In unadjusted models, participants were less likely to complete their visit if they identified as non-Hispanic Asian (0.68 [0.53–0.88]) or LGBTQIA+ (0.62 [0.52–0.74]). Female participants were more likely (1.37 [1.19–1.57]) to complete their visit. In adjusted models, participants were less likely to complete their visit if they identified as other races (0.36 [0.20–0.73]) or LGBTQIA+ (0.67 [0.52–0.86]). Participants in the 65–79 year age group were more likely (3.31 [1.90–6.33]) to complete their visit.

### Association between withdrawn status and sociodemographic characteristics

In unadjusted models, participants had a higher odds of withdrawing from the study if they were between the ages of 18–33 years (1.26 [1–13 −1.39]), identified as Non-Hispanic Black (1.27 [1.14–1.42]), Non-Hispanic Asian (1.22 [1.03–1.45]), Hispanic (1.28 [1.15–1.43]), or other races (1.91 [1.35–2.68]), identified as straight (1.39 [1.23–1.56]), had a high school education or less (1.85 [1.65–2.08]), had some college/trade school education (1.49 [1.36–1.64), had an 2019 household income of <$24,999 (1.53 [1.35–1.72]), $25,000-$49,999 (1.48 [1.31–1.6]), or $50,000 - $74,999 (1.17 [1.02–1.34]), were divorced, widowed, separated, or never married (1.21 [1.11–1.31]), did not have insurance (1.44[1.17–1.178]), were unemployed (1.40 [1.11–1.74]), be from a medically underserved area (1.29 [1.18–1.41]), reported a disability (1.37 [1.25–1.50]. Participants had a lower odds of withdrawing from the study if they were between the ages of 49–64 years (0.77 [0.69–0.85]) or 65–79 years (0.69 [0.60–0.78]) and were female (0.74 [0.68–0.81]).

Findings by age group, other races, sex assigned at birth, sexual orientation, education, and disability status were consistent in adjusted models. All other associations became non-significant.

## Discussion

In this prospective cohort study of 14,527 participants, over 85% of individuals who completed their baseline visit attended the first four follow-up visits and over 90% completed a follow-up within the last year of the study, regardless of socioeconomic or demographic factors. We observed approximately a 1–3% absolute decrease in the proportion of participants completing the second, third, and fourth follow-up visit compared to the first. There was an approximately 2% increase in the proportion of participants who completed a visit within the last year of the study compared to the first follow-up visit. Participants who were less likely to complete their first follow-up visit were non-Hispanic Black, had less than a high school education, and lived in a rural area, while those in the 49–64 age group were more likely to complete their visit. Similar trends were observed across the second, third, and fourth follow-ups, with a few notable differences. Across the several follow-ups, participants who were in the 18–33 year age group, identified as non-Hispanic Asian or other races, had some college education, and reported a disability were less likely to complete their follow-up visits. Participants in the 65–79 year age group, female participants, and those who had a household income of $75,000 - $99,999 were more likely to complete their visit. At the last follow-up visit, participants who identified as other races or LGBTQIA + and reported a disability were less likely to complete their last visit, while those in the 49–64 year and 65–79 year age groups were more likely to complete their visit. Approximately 22% of participants were withdrawn from the study, mostly due to loss to follow-up and declining to participate. Participants were more likely to be withdrawn from the study if they were in the 18–33 year age group, identified as other races or as LGBTQIA+, had less than a college degree, and reported a disability. Participants were less likely to be withdrawn from the study if they were in the 49–64 year and 65–79 year age groups and were female. Collectively, these results highlight that, despite high attendance across several follow-up visits, some groups faced challenges in staying engaged long-term in the study.

One of the primary goals of RECOVER was to engage and over-enroll underrepresented populations that represented vulnerable populations in the U.S. At baseline, our study population was composed of 57.0% White, 17.2% Hispanic, and 14.7% Black individuals, which is very similar to the U.S. population in 2020 based on the Census (57.8% of the population were White, 18.7% were Hispanic or Latino, and 12.1% were Black or African American) (14). We were able to maintain similar proportions across each follow-up visit. Additionally, we retained almost 80% of our cohort, which aligns with the retention rate of ≥ 80% that been used as a benchmark of a high retention rate (15). We achieved a high level of representation among enrolled participants, likely because of our use of several innovative methods previously shown to be effective in engaging diverse participants in research, including community-outreach advertising, faith-based health fairs, and collaboration with community health advocacy groups (15, 16). Many sites also had research staff who reflected the diversity of the study population at their sites, provided personalized follow-ups and visit reminders, and created study materials in multiple languages, which helped to increase participant engagement across racial and ethnic groups and vulnerable populations.

Our findings that minoritized populations were less likely to complete their follow-ups align with previous literature on barriers to participation in studies (17). Racial and ethnic diversity is an important aspect of a study as it increases the generalizability of the results and can uncover potential health disparities and inequities in access to healthcare and treatments (4). However, minoritized populations may be more likely to mistrust research due to historic and current mistreatment in healthcare (17). There may be apprehension to participate if they perceive the research as stigmatizing or not beneficial to their community, may impact their immigration status, that their community will be used as “lab rats”, or that informed consent and accepting the risk of unintended outcomes gives researchers the freedom to harm participants without consequences (17). This mistrust can be heightened by the lack of accessible information about participating in the research, transparent explanations on the risks and benefits to their community, and research staff that represent their community and speak their language (17). Groups that are commonly combined with other races/ethnicities due to small sample size, such as Native American, Native Hawaiian, Pacific Islander, Middle Eastern, and North African populations, may have concerns that results will misrepresent their specific community or be less motivated to participate if their community is treated as an afterthought in the study. (17) While RECOVER did incorporate tailored techniques to recruit and retain minoritized populations, such as having accessible study information in culturally competent formats and in various languages and having research staff that represented the diversity in our study population, the intensive nature of some of the medical tests may have been seen as too invasive. Some participants also may have felt that their community’s health issues were not represented or highlighted in RECOVER. Collectively, these factors could have contributed to a lower likelihood of visit completion.

Interestingly, female participants were more likely to complete their visit compared to male participants. Some researchers have attributed lower rates of visit completion among males to a lack of motivation or interest in participating, a lack of increased incentives, and scheduling conflicts (18). Survey length and intensity may also have impacted visit attendance rates, as the PASC symptom survey can be long and complex for some participants who have multiple symptoms. However, it is unclear what additional factors would motivate female participates to be more engaged in the study or why certain drawbacks did not discourage their participation compared to male participants.

We also observed that younger participants (18–33 year age group) were less likely to complete their follow-up visits while older participants (49–64 and 65–79 year age group) were more likely to complete their visits. Previous studies have reported that both younger and older participants can be difficult to retain in a study, with rates decreasing as a study progresses (19–21). Individuals in the 18–33 year age group are more likely to be in college or graduate school, be entering the workforce, or moving to different cities and states as they navigate life transitions (19). Individuals between the ages of 49–79 years are more likely to experience poor health and chronic functions that impact daily functioning (21). However, a higher proportion may also be retired and have more flexible schedules (20). These characteristics may make younger participants harder to contact and older participants more available to commit to longitudinal studies.

In our cohort, LGBTQIA+ participants were less likely to attend their last visit within the final year of the study compared to their straight counterparts. LGBTQIA+ individuals face widespread and intense social stigma that can influence their interest to participate and remain engaged in a longitudinal study where their identity is disclosed (22, 23). Identity disclosure in medical or academic settings can evoke feelings of discomfort or fear of social consequences (22). Applying an intersectional framework, forming partnerships with LGBTQIA+ community-based organizations and research advisory boards, and employing research staff who identify as LGBTQIA+ have been shown to be successful techniques in retaining LGBTQIA+ participants in studies that center LGBTQIA+ health (23). In studies that do not tailor their recruitment or retention efforts to LGBTQIA+ individuals or meaningfully engage with their community, there may be perception that the study is not beneficial to LGBTQIA+ people or that the results from the study are not reflective or representative of the community’s experiences and issues (22, 23). While RECOVER collected identity data from LGBTQIA+ participants, there has been little to no focus on reporting the unique challenges that this group faces due to COVID-19 and Long COVID. As a result, some participants may have felt disengaged as the study progressed.

It has been documented that individuals who have less education have higher attrition rates compared to their counterparts (17, 24). We also found that participants with these characteristics were less likely to complete their visits. Education level can predict health literacy and how well a participant will understand the study’s goals and procedures (24). As part of the effort to reach vulnerable and underrepresented populations, RECOVER provided study and recruitment materials in plain language and provided accessible summaries of publications and findings. However, some participants may have felt a disconnect from the study that deterred them from staying engaged long-term.

Rural participants and participants who reported a disability were also found to be less likely to complete their follow-up visits. Rural participants face complex challenges that can prevent them from maintaining long-term participation in a study. Rural participants tend to live in sparsely populated areas far from hospitals and academic institutions (25). They also are more likely to be older and have more health issues due to the lack of healthcare resources in their community (25). Completing a follow-up visit may require rural participants to travel long distances to reach the study site (25). Factors such as personal commitments and inclement weather may add an additional obstacle to completing their in-person visits and remaining engaged in the study long-term (25). For several RECOVER sites, there was transportation assistance to help offset difficulties participants experienced with traveling to the study site. Some follow-up visits could also be completed remotely. However, some rural participants may have experienced issues traveling to in-person visits, in which intensive medical tests were conducted.

People with disabilities have been historically and currently misrepresented, underrepresented, and mistreated in research and healthcare (26). The lack of competency and sensitivity for the complexities of disabilities has increased distrust in the disabled community towards research that involves disabilities (26). Participants with disabilities may have had concerns with long-term physical and communication accommodations, lack of knowledge or experience staff have on disabilities, and complications from participating that could exacerbate the severity of their disability (26). Additionally, participants who reported a disability may have struggled with the compounding impacts of their pre-existing disability and the disabling effects of COVID-19and Long COVID. While RECOVER actively included participants with disabilities in community representative panels and groups to report concerns about the study, more strategies should be developed to make participation more accessible for people with disabilities.

Despite these differences, compared with studies reporting an average retention rate of 33% for vulnerable populations, our attendance rate across all follow-ups was at least 80% for each group (27). Our high visit completion rate of vulnerable populations reflects efforts made across the sites to address issues vulnerable populations may face. Additional evidence is needed to determine why these RECOVER participants experienced difficulties completing their follow-ups so that evidence-based strategies can be designed to promote and sustain continued participation in longitudinal studies. Potential promising strategies include providing education about their health condition, establishing long-term connections with the community, reaffirming the direct benefits to participating, and navigation services to connect individuals with the care they might need, and helping individuals obtain other types of tangible benefits (e.g., disability coverage through the Social Security Administration) (15, 16). Establishing meaningful partnerships with participants beyond the scope of the research process can also help foster trust, strengthen collaboration, and reinforce a shared commitment to the study’s long-term success and impact, ultimately contributing to the advancement of the science. There is also increasing interest in the return of individual research results to promote engagement and the value of ongoing study participation (28, 29). Future studies should examine the extent to which different sites were returning individual study results or whether the manner in which the results were returned (e.g., access to electronic health records vs. discussion with a clinical provider) affected study visit completion rates.

Limitations of this study include: (1) A large portion of our cohort had a high educational attainment which signals selection bias and limits the generalizability of the study; and (2) the available data were insufficient to evaluate the effectiveness of individual strategies to promote recruitment and retention at the various sites.

The RECOVER initiative presents a critical opportunity to advance equitable research post-pandemic. Without deliberate and sustained efforts to recruit and retain a diverse, representative cohort, findings risk lacking generalizability and failing to inform care for the populations most affected by the pandemic. Future iterations of the engagement strategy should continue to prioritize equity, accessibility, and community partnerships to ensure inclusive, actionable, and ethical research outcomes. Retention and recruitment strategies must be as dynamic and adaptive as the diverse communities RECOVER aims to serve.

## Conclusion

Collectively, these results suggest that it is possible to recruit a diverse and representative cohort and retain the majority of them over time. However, there may be barriers to participating in a research study that need to be addressed in a study’s design and retention methods. It is important to listen to participants’ concerns and challenges throughout the study to better address potential difficulties and facilitate participation in the study.

## Supplementary Material

Supplementary Files

This is a list of supplementary files associated with this preprint. Click to download.

- SupplementaryMaterials06162026v3jb.docx

## Figures and Tables

**Table 1:** Sociodemographic characteristics of adult participants (N = 14,527) in the RECOVER-Adult cohort, stratified by follow-up visit completion between Oct

Characteristics	Baseline Visit		Follow-up 1 Visit Completion			Follow-up 2 Visit Completion			Follow-up 3 Visit Completion			Follow-up 4 Visit Completion		
	(N = 14,527)		(N = 14,234)			(N = 14,032)			(N = 13,842)			(N = 13,544)		
			No (n = 1,438)	Yes (n = 12,796)	p-value	No (n = 1,787)	Yes (n = 12,245)	p-value	No (n = 2,125)	Yes (n = 11,717)	p-value	No (n = 1,914)	Yes (n = 11,630)	p-value
**Age at enrollment, yr, mean[SD]**	47.1 [15.5]		44.3 [15.0]	47.4 [15.5]	< 0.001	44.2 [14.8]	47.5 [15.4]	< 0.001	44.5 [15.1]	47.5 [15.4]	< 0.001	43.0 [14.7]	47.8 [15.4]	< 0.001
**Age at enrollment**, yr, n (%)					< 0.001			< 0.001			< 0.001			< 0.001
18–33	3546 (24.4%)		437 (30.4%)	3038 (23.7%)		544 (30.4%)	2888 (23.6%)		638 (30.0%)	2743 (23.4%)		656 (34.3%)	2634 (22.6%)	
34–48	4509 (31.0%)		469 (32.6%)	3957 (30.9%)		573 (32.1%)	3790 (31.0%)		685 (32.2%)	3624 (30.9%)		611 (31.9%)	3615 (31.1%)	
49–64	4256 (29.3%)		375 (26.1%)	3787 (29.6%)		492 (27.5%)	3626 (29.6%)		571 (26.9%)	3486 (29.8%)		476 (24.9%)	3499 (30.1%)	
65–79	2070 (14.2%)		142 (9.9%)	1888 (14.8%)		159 (8.9%)	1828 (14.9%)		207 (9.7%)	1757 (15.0%)		154 (8.0%)	1773 (15.2%)	
80+	145 (1.0%)		15 (1.0%)	125 (1.0%)		18 (1.0%)	113 (0.9%)		23 (1.1%)	107 (0.9%)		17 (0.9%)	109 (0.9%)	
Missing	1 (0.0%)		0 (0%)	1 (0.0%)		1 (0.1%)	0 (0%)		1 (0.0%)	0 (0%)		0 (0%)	0 (0%)	
**Race/Ethnicity**, n (%)					< 0.001			< 0.001			< 0.001			< 0.001
Non-Hispanic White	8280 (57.0%)		673 (46.8%)	7453 (58.2%)		847 (47.4%)	7181 (58.6%)		1033 (48.6%)	6901 (58.9%)		937 (49.0%)	6840 (58.8%)	
Non-Hispanic Black	2129 (14.7%)		285 (19.8%)	1791 (14.0%)		312 (17.5%)	1725 (14.1%)		364 (17.1%)	1633 (13.9%)		312 (16.3%)	1636 (14.1%)	
Non-Hispanic Asian	846 (5.8%)		82 (5.7%)	751 (5.9%)		125 (7.0%)	702 (5.7%)		150 (7.1%)	669 (5.7%)		142 (7.4%)	662 (5.7%)	
Hispanic	2496 (17.2%)		307 (21.3%)	2126 (16.6%)		385 (21.5%)	2005 (16.4%)		424 (20.0%)	1931 (16.5%)		382 (20.0%)	1915 (16.5%)	
Two or more races	500 (3.4%)		50 (3.5%)	443 (3.5%)		57 (3.2%)	425 (3.5%)		88 (4.1%)	388 (3.3%)		78 (4.1%)	391 (3.4%)	
Other races	156 (1.1%)		24 (1.7%)	131 (1.0%)		37 (2.1%)	115 (0.9%)		38 (1.8%)	111 (0.9%)		34 (1.8%)	111 (1.0%)	
Missing	120 (0.8%)		17 (1.2%)	101 (0.8%)		24 (1.3%)	92 (0.8%)		28 (1.3%)	84 (0.7%)		29 (1.5%)	75 (0.6%)	
**Sex assigned at birth**, n (%)					0.17			0.004			< 0.001			0.004
Female/Intersex	10466 (72.0%)		1015 (70.6%)	9257 (72.3%)		1235 (69.1%)	8899 (72.7%)		1472 (69.3%)	8553 (73.0%)		1330 (69.5%)	8510 (73.2%)	
Male	4022 (27.7%)		419 (29.1%)	3504 (27.4%)		541 (30.3%)	3319 (27.1%)		642 (30.2%)	3140 (26.8%)		569 (29.7%)	3103 (26.7%)	
Missing	39 (0.3%)		4 (0.3%)	35 (0.3%)		11 (0.6%)	27 (0.2%)		11 (0.5%)	24 (0.2%)		15 (0.8%)	17 (0.1%)	
**Sexual orientation**, n (%)					0.22			0.57			0.02			< 0.001
LGBTQIA+	1586 (10.9%)		170 (11.8%)	1384 (10.8%)		202 (11.3%)	1332 (10.9%)		260 (12.2%)	1245 (10.6%)		247 (12.9%)	1220 (10.5%)	
Straight	12251 (84.3%)		1189 (82.7%)	10823 (84.6%)		1494 (83.6%)	10352 (84.5%)		1739 (81.8%)	9963 (85.0%)		1547 (80.8%)	9912 (85.2%)	
Missing	690 (4.7%)		79 (5.5%)	589 (4.6%)		91 (5.1%)	561 (4.6%)		126 (5.9%)	509 (4.3%)		120 (6.3%)	498 (4.3%)	
**Education**, n (%)					< 0.001			< 0.001			< 0.001			< 0.001
Highschool or less		1706 (11.7%)	295 (20.5%)	1350 (10.6%)		337 (18.9%)	1270 (10.4%)		352 (16.6%)	1205 (10.3%)		308 (16.1%)	1188 (10.2%)	
Some college/trade school		3373 (23.2%)	353 (24.5%)	2918 (22.8%)		464 (26.0%)	2762 (22.6%)		544 (25.6%)	2629 (22.4%)		497 (26.0%)	2593 (22.3%)	
College degree or higher		9294 (64.0%)	770 (53.5%)	8397 (65.6%)		958 (53.6%)	8095 (66.1%)		1194 (56.2%)	7775 (66.4%)		1065 (55.6%)	7754 (66.7%)	
Missing		154 (1.1%)	20 (1.4%)	131 (1.0%)		28 (1.6%)	118 (1.0%)		35 (1.6%)	108 (0.9%)		44 (2.3%)	95 (0.8%)	
**Household Income in 2019**, n (%)					< 0.001			< 0.001			< 0.001			< 0.001
<$24,999		2216 (15.3%)	281 (19.5%)	1883 (14.7%)		348 (19.5%)	1768 (14.4%)		359 (16.9%)	1712 (14.6%)		336 (17.6%)	1671 (14.4%)	
$25,000-$49,999		2023 (13.9%)	226 (15.7%)	1749 (13.7%)		267 (14.9%)	1672 (13.7%)		301 (14.2%)	1601 (13.7%)		273 (14.3%)	1579 (13.6%)	
$50,000 - $74,999		1828 (12.6%)	159 (11.1%)	1631 (12.7%)		202 (11.3%)	1564 (12.8%)		245 (11.5%)	1506 (12.9%)		232 (12.1%)	1473 (12.7%)	
$75,000 - $99,999		1625 (11.2%)	126 (8.8%)	1477 (11.5%)		144 (8.1%)	1440 (11.8%)		221 (10.4%)	1354 (11.6%)		174 (9.1%)	1376 (11.8%)	
$100,000+		5246 (36.1%)	410 (28.5%)	4768 (37.3%)		530 (29.7%)	4601 (37.6%)		669 (31.5%)	4406 (37.6%)		584 (30.5%)	4410 (37.9%)	
Missing		1589 (10.9%)	236 (16.4%)	1288 (10.1%)		296 (16.6%)	1200 (9.8%)		330 (15.5%)	1138 (9.7%)		315 (16.5%)	1121 (9.6%)	
**Marital status**, n (%)					0.002			< 0.001			0.15			< 0.001
Divorced, widowed, separated, or never married		5263 (36.2%)	548 (38.1%)	4612 (36.0%)		678 (37.9%)	4391 (35.9%)		757 (35.6%)	4226 (36.1%)		720 (37.6%)	4145 (35.6%)	
Married or living with partner		8606 (59.2%)	758 (52.7%)	7696 (60.1%)		940 (52.6%)	7414 (60.5%)		1178 (55.4%)	7083 (60.5%)		1004 (52.5%)	7090 (61.0%)	
Missing		658 (4.5%)	132 (9.2%)	488 (3.8%)		169 (9.5%)	440 (3.6%)		190 (8.9%)	408 (3.5%)		190 (9.9%)	395 (3.4%)	
**Insurance status**, n (%)					0.07			0.008			0.27			0.59
Yes		13310 (91.6%)	1234 (85.8%)	11850 (92.6%)		1529 (85.6%)	11378 (92.9%)		1842 (86.7%)	10902 (93.0%)		1654 (86.4%)	10830 (93.1%)	
No		450 (3.1%)	52 (3.6%)	376 (2.9%)		68 (3.8%)	350 (2.9%)		67 (3.2%)	338 (2.9%)		56 (2.9%)	335 (2.9%)	
Missing		767 (5.3%)	152 (10.6%)	570 (4.5%)		190 (10.6%)	517 (4.2%)		216 (10.2%)	477 (4.1%)		204 (10.7%)	465 (4.0%)	
**Employment status**, n (%)					< 0.001			< 0.001			< 0.001			< 0.001
Employed		9231 (63.5%)	891 (62.0%)	8184 (64.0%)		1102 (61.7%)	7865 (64.2%)		1322 (62.2%)	7538 (64.3%)		1177 (61.5%)	7487 (64.4%)	
Unemployed		410 (2.8%)	51 (3.5%)	345 (2.7%)		62 (3.5%)	323 (2.6%)		64 (3.0%)	312 (2.7%)		60 (3.1%)	304 (2.6%)	
Missing		450 (3.1%)	106 (7.4%)	310 (2.4%)		132 (7.4%)	274 (2.2%)		145 (6.8%)	253 (2.2%)		146 (7.6%)	243 (2.1%)	
**From a medically underserved area**, n (%)					< 0.001			0.05			0.35			0.15
No		10766 (74.1%)	1007 (70.0%)	9554 (74.7%)		1290 (72.2%)	9113 (74.4%)		1558 (73.3%)	8707 (74.3%)		1392 (72.7%)	8643 (74.3%)	
Yes		3761 (25.9%)	431 (30.0%)	3242 (25.3%)		497 (27.8%)	3132 (25.6%)		567 (26.7%)	3010 (25.7%)		522 (27.3%)	2987 (25.7%)	
Missing		0 (0%)	0 (0%)	0 (0%)		0 (0%)	0 (0%)		0 (0%)	0 (0%)		0 (0%)	0 (0%)	
**Primary language**, n (%)					0.001			< 0.001			0.001			0.006
English		13049 (89.8%)	1201 (83.5%)	11611 (90.7%)		1493 (83.5%)	11148 (91.0%)		1799 (84.7%)	10669 (91.1%)		1605 (83.9%)	10601 (91.2%)	
Non-English		992 (6.8%)	122 (8.5%)	850 (6.6%)		148 (8.3%)	804 (6.6%)		174 (8.2%)	771 (6.6%)		151 (7.9%)	768 (6.6%)	
Missing		486 (3.3%)	115 (8.0%)	335 (2.6%)		146 (8.2%)	293 (2.4%)		152 (7.2%)	277 (2.4%)		158 (8.3%)	261 (2.2%)	
**Living in a rural area**, n (%)					< 0.001			0.001			0.02			0.22
No		13049 (89.8%)	1351 (94.0%)	12268 (95.9%)		1683 (94.2%)	11739 (95.9%)		2012 (94.7%)	11230 (95.8%)		1819 (95.0%)	11128 (95.7%)	
Yes		992 (6.8%)	87 (6.1%)	528 (4.1%)		104 (5.8%)	506 (4.1%)		113 (5.3%)	487 (4.2%)		95 (5.0%)	502 (4.3%)	
Missing		0 (0%)	0 (0%)	0 (0%)		0 (0%)	0 (0%)		0 (0%)	0 (0%)		0 (0%)	0 (0%)	
**Disability status**, n (%)					< 0.001			< 0.001			< 0.001			< 0.001
No		10507 (72.3%)	935 (65.0%)	9417 (73.6%)		1147 (64.2%)	9080 (74.2%)		1398 (65.8%)	8702 (74.3%)		1243 (64.9%)	8657 (74.4%)	
Yes		3165 (21.8%)	341 (23.7%)	2735 (21.4%)		438 (24.5%)	2578 (21.1%)		491 (23.1%)	2472 (21.1%)		450 (23.5%)	2437 (21.0%)	
Missing		855 (5.9%)	162 (11.3%)	644 (5.0%)		202 (11.3%)	587 (4.8%)		236 (11.1%)	543 (4.6%)		221 (11.5%)	536 (4.6%)	

**Table 2:** Association between follow-up visit completion and demographic characteristics among adult participants (n = 14,527) in the RECOVER-Adult cohort between October 2021 – October 2025

Characteristics	Follow-up 1 Visit Completion		Follow-up 2 Visit Completion		Follow-up 3 Visit Completion		Follow-up 4 Visit Completion		Last Visit Completion	
	Unadjusted	Adjusted	Unadjusted	Adjusted	Unadjusted	Adjusted	Unadjusted	Adjusted	Unadjusted	Adjusted
	OR	OR^d^	OR	OR^d^	OR	OR^d^	OR	OR^d^	OR	OR^d^
	(95% CI)	(95% CI)	(95% CI)	(95% CI)	(95% CI)	(95% CI)	(95% CI)	(95% CI)	(95% CI)	(95% CI)
**Age at enrollment, yr** (ref = 34–48)										
18–33	0.82 (0.72–0.95)	0.83 (0.68–1.00)	0.80 (0.71–0.91)	0.74 (0.62–0.88)	0.81 (0.72–0.91)	0.85 (0.72–1.00)	0.68 (0.60–0.77)	0.69 (0.58–0.81)	0.76 (0.65–0.89)	0.83 (0.66–1.03)
49–64	1.20 (1.04–1.38)	1.39 (1.14–1.69)	1.11 (0.98–1.27)	1.28 (1.08–1.53)	1.15 (1.02–1.30)	1.27 (1.08–1.49)	1.24 (1.09–1.41)	1.38 (1.16–1.64)	1.30 (1.10–1.54)	1.34 (1.08–1.68)
65–79	1.58 (1.30–1.92)	1.43 (1.00–2.11)	1.74 (1.45–2.10)	1.51 (1.09–2.16)	1.60 (1.36–1.90)	1.65 (1.21–2.31)	1.95 (1.62–2.35)	1.78 (1.27–2.57)	2.29 (1.78–2.98)	3.31 (1.90–6.33)
80+	0.99 (0.59–1.77)	1.30 (0.26–23.78)	0.95 (0.59–1.62)	1.84 (0.36–33.51)	0.88 (0.57–1.42)	0.62 (0.19–2.76)	1.08 (0.66–1.88)	2.10 (0.41–38.33)	1.14 (0.60–2.43)	-
**Race/Ethnicity** (ref = Non-Hispanic White)										
Non-Hispanic Black	0.57 (0.49–0.66)	0.70 (0.55–0.89)	0.65 (0.57–0.75)	0.90 (0.72–1.13)	0.67 (0.59–0.77)	0.77 (0.63–0.96)	0.72 (0.63–0.83)	0.77 (0.62–0.95)	0.85 (0.70–1.03)	0.89 (0.67–1.21)
Non-Hispanic Asian	0.83 (0.65–1.06)	1.20 (0.85–1.76)	0.66 (0.54–0.81)	0.87 (0.66–1.17)	0.67 (0.55–0.81)	0.73 (0.57–0.95)	0.64 (0.53–0.78)	0.71 (0.55–0.93)	0.68 (0.53–0.88)	0.73 (0.52–1.03)
Hispanic	0.63 (0.54–0.72)	0.81 (0.65–1.01)	0.61 (0.54–0.70)	0.96 (0.78–1.18)	0.68 (0.60–0.77)	0.92 (0.76–1.11)	0.69 (0.60–0.78)	0.91 (0.74–1.11)	0.77 (0.65–0.91)	0.97 (0.75–1.28)
Two or more races	0.80 (0.60–1.10)	0.95 (0.63–1.49)	0.88 (0.67–1.18)	1.38 (0.91–2.18)	0.66 (0.52–0.84)	0.87 (0.62–1.26)	0.69 (0.54–0.89)	1.03 (0.71–1.55)	0.83 (0.59–1.19)	1.04 (0.65–1.77)
Other races	0.49 (0.32–0.79)	0.77 (0.41–1.62)	0.37 (0.25–0.54)	0.40 (0.24–0.67)	0.44 (0.30–0.64)	0.51 (0.31–0.88)	0.45 (0.31–0.67)	0.56 (0.33–0.99)	0.49 (0.30–0.86)	0.36 (0.20–0.73)
**Sex assigned at birth** (ref = Male)	1.09 (0.97–1.23)	1.10 (0.92–1.30)	1.17 (1.05–1.31)	1.22 (1.04–1.42)	1.19 (1.07–1.31)	1.22 (1.06–1.41)	1.17 (1.05–1.30)	1.30 (1.12–1.51)	1.37 (1.19–1.57)	1.17 (0.95–1.42)
**Sexual orientation** (ref = Straight)	0.89 (0.76–1.06)	0.95 (0.76–1.20)	0.95 (0.81–1.12)	1.01 (0.82–1.25)	0.84 (0.73–0.97)	0.86 (0.71–1.04)	0.77 (0.67–0.89)	0.91 (0.75–1.12)	0.62 (0.52–0.74)	0.67 (0.52–0.86)
**Education** (ref = College degree of higher)										
Highschool or less	0.42 (0.36–0.49)	0.49 (0.38–0.65)	0.45 (0.39–0.51)	0.59 (0.46–0.77)	0.53 (0.46–0.60)	0.59 (0.46–0.76)	0.53 (0.46–0.61)	0.61 (0.47–0.80)	0.65 (0.54–0.79)	0.83 (0.57–1.22)
Some college/trade school	0.76 (0.66–0.87)	0.88 (0.72–1.08)	0.70 (0.63–0.79)	0.81 (0.68–0.98)	0.74 (0.66–0.83)	0.75 (0.63–0.88)	0.72 (0.64–0.80)	0.85 (0.71–1.02)	0.79 (0.68–0.93)	0.84 (0.66–1.07)
**Household Income in 2019** (ref = $100,000+)										
<$24,999	0.58 (0.49–0.68)	1.01 (0.75–1.37)	0.59 (0.51–0.68)	0.93 (0.71–1.21)	0.72 (0.63–0.83)	1.15 (0.88–1.49)	0.66 (0.57–0.76)	1.17 (0.89–1.53)	0.83 (0.68–1.01)	1.28 (0.89–1.86)
$25,000-$49,999	0.67 (0.56–0.79)	0.95 (0.74–1.22)	0.72 (0.62–0.85)	1.14 (0.90–1.44)	0.81 (0.70–0.94)	1.18 (0.95–1.47)	0.77 (0.66–0.90)	1.14 (0.91–1.43)	0.88 (0.72–1.09)	1.06 (0.79–1.42)
$50,000 - $74,999	0.88 (0.73–1.07)	1.08 (0.85–1.39)	0.89 (0.75–1.06)	1.07 (0.86–1.33)	0.93 (0.80–1.09)	1.06 (0.87–1.30)	0.84 (0.72–0.99)	1.13 (0.92–1.41)	1.08 (0.87–1.35)	1.17 (0.89–1.56)
$75,000 - $99,999	1.01 (0.82–1.25)	1.16 (0.90–1.50)	1.15 (0.95–1.40)	1.54 (1.21–1.97)	0.93 (0.79–1.10)	1.11 (0.90–1.36)	1.05 (0.88–1.26)	1.21 (0.98–1.51)	1.38 (1.08–1.78)	1.33 (1.00–1.79)
**Marital status** (ref = Married or living with a partner)	0.83 (0.74–0.93)	0.98 (0.82–1.16)	0.82 (0.74–0.91)	0.91 (0.78–1.06)	0.93 (0.84–1.03)	1.06 (0.91–1.23)	0.82 (0.74–0.90)	0.91 (0.78–1.06)	0.88 (0.77–1.01)	0.98 (0.80–1.20)
**Insurance** (ref = yes)	0.75 (0.57–1.02)	1.13 (0.75–1.77)	0.69 (0.53–0.91)	1.00 (0.69–1.49)	0.85 (0.66–1.12)	1.11 (0.76–1.67)	0.91 (0.69–1.23)	1.15 (0.77–1.76)	0.83 (0.58–1.22)	0.78 (0.49–1.31)
**Employment status** (ref = Employed)	0.74 (0.55–1.01)	0.96 (0.67–1.41)	0.73 (0.56–0.97)	0.89 (0.64–1.25)	0.85 (0.65–1.13)	1.04 (0.75–1.48)	0.80 (0.60–1.07)	0.90 (0.64–1.27)	0.73 (0.52–1.06)	0.71 (0.48–1.08)
**From a medically underserved area** (ref = No)	0.79 (0.70–0.89)	1.02 (0.85–1.22)	0.89 (0.80–1.00)	1.15 (0.97–1.36)	0.95 (0.86–1.06)	1.09 (0.93–1.27)	0.92 (0.83–1.03)	1.13 (0.96–1.33)	0.89 (0.77–1.03)	1.12 (0.90–1.40)
**Primary language** (ref = English)	0.72 (0.59–0.88)	0.95 (0.71–1.30)	0.73 (0.61–0.88)	0.79 (0.61–1.04)	0.75 (0.63–0.89)	0.95 (0.74–1.24)	0.77 (0.64–0.93)	0.98 (0.75–1.30)	0.86 (0.67–1.11)	1.02 (0.72–1.48)
**Living in a rural area** (ref = No)	0.67 (0.53–0.85)	0.61 (0.44–0.88)	0.70 (0.56–0.87)	0.56 (0.41–0.77)	0.77 (0.63–0.96)	0.65 (0.48–0.89)	0.86 (0.69–1.09)	0.73 (0.53–1.03)	1.07 (0.78–1.50)	0.97 (0.61–1.64)
**Disability status** (ref = No)	0.80 (0.70–0.91)	0.87 (0.72–1.06)	0.74 (0.66–0.84)	0.84 (0.71–1.01)	0.81 (0.72–0.91)	0.84 (0.71–0.99)	0.78 (0.69–0.87)	0.84 (0.71–1.00)	0.79 (0.68–0.92)	0.78 (0.63–0.99)

d adjusted model included age at enrollment, race/ethnicity, sex assigned at birth, sexual orientation, education, income, marital status, insurance status, employment status, from a medically underserved area, primary language, living in a rural area, and disability status

**Table 3:** Sociodemographic characteristics of adult participants (N = 14,527) in the RECOVER-Adult cohort, stratified by withdrawn status

	Withdrawn Status		
	No (N = 11,381)	Yes (N = 3,149)	p-value
Characteristics			
**Age at enrollment, yr, mean[SD]**	47.8 [15.3]	44.6 [15.6]	< 0.001
**Age at enrollment**, yr, n (%)			< 0.001
18–33	2565 (22.5%)	946 (30.0%)	
34–48	3471 (30.5%)	1020 (32.4%)	
49–64	3455 (30.4%)	779 (24.7%)	
65–79	1720 (15.1%)	347 (11.0%)	
80+	104 (0.9%)	41 (1.3%)	
Missing	66 (0.6%)	16 (0.5%)	
**Race/Ethnicity**, n (%)			< 0.001
Non-Hispanic White	6645 (58.4%)	1637 (52.0%)	
Non-Hispanic Black	1621 (14.2%)	508 (16.1%)	
Non-Hispanic Asian	650 (5.7%)	196 (6.2%)	
Hispanic	1896 (16.7%)	600 (19.1%)	
Two or more races	388 (3.4%)	112 (3.6%)	
Other races	106 (0.9%)	50 (1.6%)	
Missing	75 (0.7%)	46 (1.5%)	
**Sex assigned at birth**, n (%)			< 0.001
Female/Intersex	8360 (73.5%)	2108 (66.9%)	
Male	3002 (26.4%)	1021 (32.4%)	
Missing	19 (0.2%)	20 (0.6%)	
**Sexual orientation**, n (%)			< 0.001
LGBTQIA+	9718 (85.4%)	2534 (80.5%)	
Straight	1165 (10.2%)	421 (13.4%)	
Missing	498 (4.4%)	194 (6.2%)	
**Education**, n (%)			< 0.001
Highschool or less	1198 (10.5%)	508 (16.1%)	
Some college/trade school	2514 (22.1%)	859 (27.3%)	
College degree or higher	7563 (66.5%)	1732 (55.0%)	
Missing	106 (0.9%)	50 (1.6%)	
**Household Income in 2019**, n (%)			< 0.001
<$24,999	1654 (14.5%)	562 (17.8%)	
$25,000-$49,999	1522 (13.4%)	501 (15.9%)	
$50,000 - $74,999	1450 (12.7%)	378 (12.0%)	
$75,000 - $99,999	1327 (11.7%)	298 (9.5%)	
$100,000+	4291 (37.7%)	956 (30.4%)	
Missing	1137 (10.0%)	454 (14.4%)	
**Marital status**, n (%)			< 0.001
Divorced, widowed, separated, or never married	4049 (35.6%)	1214 (38.6%)	
Married or living with partner	6893 (60.6%)	1714 (54.4%)	
Missing	439 (3.9%)	221 (7.0%)	
**Insurance status**, n (%)			0.008
Yes	10561 (92.8%)	2750 (87.3%)	
No	327 (2.9%)	123 (3.9%)	
Missing	493 (4.3%)	276 (8.8%)	
**Employment status**, n (%)			< 0.001
Employed	7293 (64.1%)	1939 (61.6%)	
Unemployed	299 (2.6%)	111 (3.5%)	
Missing	283 (2.5%)	169 (5.4%)	
**From a medically underserved area**, n (%)			< 0.001
No	8558 (75.2%)	2210 (70.2%)	
Yes	2823 (24.8%)	939 (29.8%)	
Missing	0 (0%)	0 (%)	
**Primary language**, n (%)			0.169
English	10312 (90.6%)	2738 (86.9%)	
Non-English	765 (6.7%)	227 (7.2%)	
Missing	304 (2.7%)	184 (5.8%)	
**Living in a rural area**, n (%)			0.883
No	10895 (95.7%)	3012 (95.6%)	
Yes	486 (4.3%)	137 (4.4%)	
Missing	0 (0%)	0 (%)	
**Disability status**, n (%)			< 0.001
No	8439 (74.1%)	2069 (65.7%)	
Yes	2370 (20.8%)	795 (25.2%)	
Missing	572 (5.0%)	285 (9.1%)	

**Table 4:** Association between withdrawn status and demographic characteristics among adult participants (n = 14,527) in the RECOVER-Adult cohort between October 2021 – October 2025

Characteristics	Withdrawn Status	
	Unadjusted OR (95% CI)	Adjusted OR^a^
		(95% CI)
**Age at enrollment, yr** (ref = 34–48)		
18–33	1.26 (1.13–1.39)	1.22 (1.06–1.40)
49–64	0.77 (0.69–0.85)	0.65 (0.56–0.74)
65–79	0.69 (0.60–0.78)	0.68 (0.52–0.87)
80+	1.34 (0.92–1.92)	0.56 (0.09–2.07)
**Race/Ethnicity** (ref = Non-Hispanic White)		
Non-Hispanic Black	1.27 (1.14–1.42)	0.96 (0.80–1.15)
Non-Hispanic Asian	1.22 (1.03–1.45)	1.04 (0.82–1.32)
Hispanic	1.28 (1.15–1.43)	1.00 (0.85–1.18)
Two or more races	1.17 (0.94–1.45)	0.92 (0.67–1.25)
Other races	1.91 (1.35–2.68)	2.16 (1.36–3.39)
**Sex assigned at birth** (ref = Male)	0.74 (0.68–0.81)	0.73 (0.65–0.83)
**Sexual orientation** (ref = Straight)	1.39 (1.23–1.56)	1.25 (1.07–1.47)
**Education** (ref = College degree of higher)		
Highschool or less	1.85 (1.65–2.08)	1.83 (1.48–2.26)
Some college/trade school	1.49 (1.36–1.64)	1.46 (1.26–1.68)
**Household Income in 2019** (ref = $100,000+)		
<$24,999	1.53 (1.35–1.72)	0.85 (0.68–1.06)
$25,000-$49,999	1.48 (1.31–1.67)	0.98 (0.81–1.17)
$50,000 - $74,999	1.17 (1.02–1.34)	0.93 (0.78–1.10)
$75,000 - $99,999	1.01 (0.87–1.16)	0.86 (0.72–1.02)
**Marital status** (ref = Married or living with a partner)	1.21 (1.11–1.31)	1.04 (0.92–1.18)
**Insurance status** (ref = yes)	1.44 (1.17–1.78)	1.32 (0.98–1.77)
**Employment status** (ref = Employed)	1.40 (1.11–1.74)	1.17 (0.89–1.53)
**From a medically underserved area** (ref = No)	1.29 (1.18–1.41)	1.11 (0.97–1.26)
**Primary language** (ref = English)	1.12 (0.96–1.30)	0.90 (0.72–1.14)
**Living in a rural area** (ref = No)	1.02 (0.84–1.23)	1.03 (0.77–1.37)
**Disability status** (ref = No)	1.37 (1.25–1.50)	1.27 (1.10–1.46)

a adjusted model included age at enrollment, race/ethnicity, sex assigned at birth, sexual orientation, education, income, marital status, insurance status, employment status, from a medically underserved area, primary language, living in a rural area, and disability status

## Data Availability

RECOVER-Adult cohort data is publicly available at https://recovercovid.org/data. De-identified participant level data can be accessed following the submission and approval of a research proposal and a signed data access agreement.
